# Coexistence of acute appendicitis and cecal inflammatory pseudotumor in a young male: Rare collision at the cecum – A case study

**DOI:** 10.1016/j.ijscr.2025.111230

**Published:** 2025-03-28

**Authors:** Abdinasir Mohamed Mohamud, Hassan Elmi Moumin, Ahmed Suleiman, Isaq Omer, Roukia Mahamad Nour, Abdirahman Omer Ali

**Affiliations:** a Surgery department, Jigjiga University, Ethiopia; b Pathology department, Jigjiga University, Ethiopia; c College of Health Sciences, School of Medicine and Surgery, Amoud University, Borama, Somalia; d Borama Regional Hospital, Surgery department, Borama, Somalia

**Keywords:** Acute appendicitis, Inflammatory Pseudotumor, Surgical exploration, Histopathology, Abdominal pain

## Abstract

**Introduction and importance:**

Inflammatory pseudotumors (IPTs) of the cecum are rare benign lesions that can pose significant diagnostic challenges due to their ability to mimic malignancy. The occurrence of a cecal IPT in conjunction with acute appendicitis is an exceedingly uncommon phenomenon, with scant documentation in existing literature. Understanding these conditions is crucial for timely and accurate diagnosis.

**Case presentation:**

We present a case involving a 24-year-old male who exhibited classic symptoms of acute appendicitis. During intraoperative exploration, an inflamed appendix was found alongside a 4x4x3 cm inflammatory mass located on the cecum. The patient underwent a successful resection of the mass and appendectomy. Histopathological examination confirmed the diagnoses of both acute appendicitis and an inflammatory pseudotumor of the cecum.

**Clinical discussion:**

This case underscores the necessity of maintaining a broad differential diagnosis when patients present with acute abdominal pain. The rare coexistence of acute appendicitis and a cecal IPT highlights the importance of thorough surgical exploration and histopathological evaluation to achieve an accurate diagnosis and appropriate management. The findings contribute to the limited literature on this subject and emphasize the potential for atypical presentations in common surgical emergencies.

**Conclusion:**

In conclusion, this case illustrates the importance of vigilance in diagnosing abdominal conditions that may present similarly. The coexistence of acute appendicitis and a cecal IPT serves as a reminder of the complexities involved in surgical diagnosis, advocating for comprehensive evaluation strategies.

## Highlights

• **Rare Co-occurrence:** Reports an extremely rare co-occurrence of acute appendicitis and an inflammatory pseudotumor (IPT) of the cecum in a young male.
• **Diagnostic Challenges:** Highlights the difficulties encountered in differentiating between common and rare pathologies presenting with similar symptoms of acute abdominal pain.
• **Importance of Surgical Exploration:** Emphasizes the importance of thorough intraoperative exploration in identifying unexpected pathologies, such as cecal IPTs.
• **Histopathological Role:** Underscores the critical role of histopathological analysis in accurate diagnosis and differentiation of benign from malignant lesions.
• **Broader Differentials in Acute Abdominal Pain:** Demonstrates the need to consider broader differentials in acute abdominal pain and avoid premature diagnostic closure.

## 1 Introduction

Inflammatory pseudo-tumors (IPTs) are rare lesions, accounting for less than 0.0001 % of the general population [1]. While historically described as neoplasms, IPTs are now primarily considered reactive or inflammatory lesions rather than true neoplasms, representing an exaggerated response to injury or inflammation [2,18]. IPT is a unique lesion with uncertain etiopathogenesis. Although it has been referred to with several names, such as plasma cell granuloma, inflammatory pseudotumor, and myxoid hamartoma, the lesion was first described by Bahadori and Liebow in 1973 as Inflammatory Myofibroblastic Tumor (IMT) [2]. Inflammatory pseudotumor (IPT) is a reactive condition which occurs in many organs. They may occur in any anatomical location. Although IPTs can occur at any age, they are most commonly described in children and young adults. Due to their often vague and inconclusive clinical presentation, IPTs must be differentiated from infectious, granulomatous, autoimmune, and malignant lesions [3] [4]. Alimentary tract IPTs were first described in the stomach; however, they remain exceedingly rare, particularly in the colon, where their etiology remains unclear. IPTs are radiologically indistinguishable from malignant lesions, which can result in unnecessary oncological resections [1]. The coexistence of acute appendicitis and a cecal IPT is exceptionally rare. This case report adds to the limited literature on this unusual clinical entity. This case is presented in accordance with the SCARE 2023 guidelines [5].

## 2 Case presentation

A 24-year-old male presented to our surgical emergency department (ER) with a one-day history of right lower quadrant abdominal pain. The pain was initially periumbilical, then migrated to the right lower quadrant. He also reported anorexia, nausea, and three episodes of non-bilious vomiting, along with low-grade intermittent fever. There was no urinary complaint, jaundice, or changes in bowel habits. He had no prior medical or surgical history. He works at a local non-governmental organization (NGO) and reports daily khat chewing for the past five years, without a history of smoking or alcohol use.

On examination, he was in mild distress and appeared acutely ill. His vital signs were within the normal ranges (blood pressure 110/70 mmHg, pulse rate 88 bpm, temperature 36.2 °C, respiratory rate 18 breaths per minute, and oxygen saturation 99 % on room air). Abdominal examination revealed right lower quadrant direct and rebound tenderness, positive Rovsing's sign, and a positive Psoas sign, without guarding or rigidity. Other systemic examinations were unremarkable. An Alvarado score was calculated to be 6, indicating a high probability of appendicitis. The following preoperative investigations were performed:

**Table:** 

Investigation	Result	Normal Range
White Blood Cell Count (WBC)	6700/μL	4000 - 11,000/μL
Neutrophils	72 %	40–70 %
Hemoglobin	14.4 g/dL	13.5–17.5 g/dL
Platelets	268,000/μL	150,000 - 450,000/μL
Abdominal Ultrasound	Abdominal ultrasound findings were inconclusive, as the appendix was difficult to visualize. However, a 3 × 3 cm fluid collection with surrounding inflammation was identified in the right lower quadrant.	Normal Appendix, No fluid collection

Given the clinical picture and imaging findings**,** acute appendicitis with possible peri appendiceal fluid collection was considered. Due to the clinical picture, Alvarado score, the identification of a fluid collection suggesting possible abscess formation, limited resources, the desire for a definitive diagnosis, and the lack of immediate CT scan availability in our facility, a contrast-enhanced CT (CECT) scan was not performed. Unfortunately, the ultrasound image is unavailable for inclusion in this report (Pictures were not taken on initial encounter). Given the patient's relatively short symptom duration and the lack of expertise in image-guided drainage of potential abscesses in our center, conservative treatment was not pursued. In our practice, appendectomy is generally preferred for patients presenting with suspected appendicitis of short duration. Prior to surgery, the patient and his family were thoroughly counselled regarding the planned procedure, the expected outcomes, potential challenges, and possible complications. Informed consent was then obtained.

**Fig. 1:**
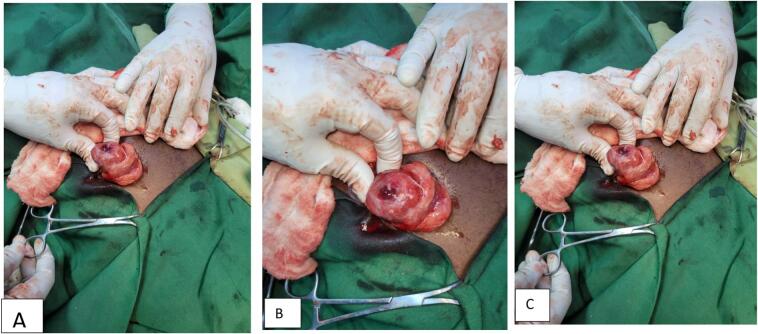
The image appears to show an inflamed appendix with a pseudotumor, characterized by swollen, abnormal tissue.

After adequate resuscitation and obtaining informed consent, a McBurney incision was made. Intraoperative findings revealed approximately 20 mL of reactive fluid in the right lower quadrant area. A 4x4x3 cm circular mass was identified on the anterior wall of the cecum and ascending colon, predominantly on the mesenteric side. This mass had a central umbilication communicating with the right lower quadrant fluid. The appendix was grossly inflamed, retrocecally located and separate from the mass (Fig. 1). The incision was extended vertically to improve exposure and facilitate right colon mobilization. The mass was completely excised via a right hemicolectomy, and small serosal tears were repaired with 3–0 absorbable Vicryl sutures. The surgical field was lavaged with warm normal saline before wound closure.

## 3 Postoperative course and histopathology

**Fig. 2:**
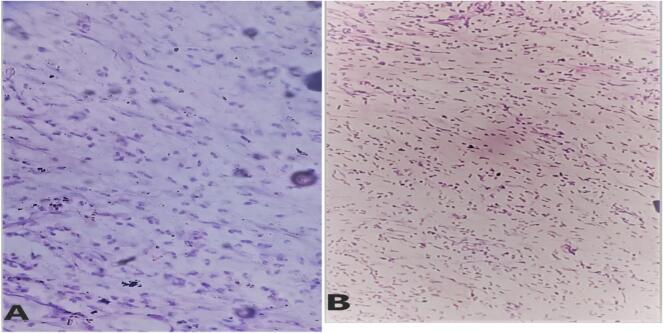
A &B: (40×) Histologic section showing fibroblast like spindle cells along with myofibroblasts admixed with numerous lymphocytes, plasma cells, neutrophils and eosinophil.

**Fig. 3:**
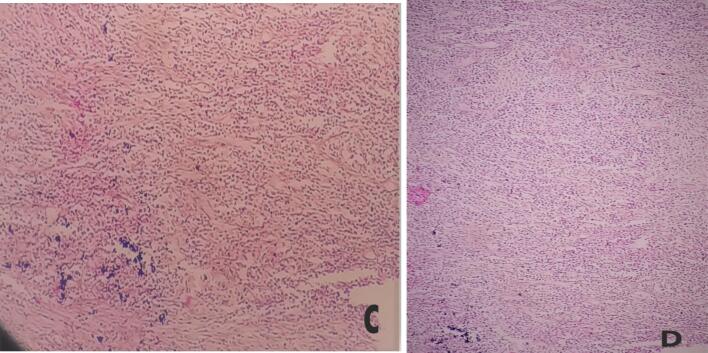
C (40×) & D (20×) Histologic section having dense mixed inflammation predominantly of sheets of neutrophils and eosinophils interspersed between granulation-like tissue composed of fibroblasts and variable sized capillary channels with no specific patterns.

The patient tolerated the procedure well, was extubated successfully, and transferred to the post-anesthesia care unit (PACU) in stable condition. Postoperatively, he received intravenous broad-spectrum antibiotics, analgesia, and intravenous fluids. His recovery was uneventful, and he was discharged on postoperative day five. At his 10-day follow-up, the patient was symptom-free, his wound had healed well, and stitches were removed. He is currently being followed regularly with clinical history, physical examination and imaging to monitor for any signs of recurrence. Currently, his wound is completely healed, and he has no complaints. Histopathological examination of the excised specimens confirmed acute appendicitis and an inflammatory pseudotumor (IPT). The IPT was composed of spindle cells, inflammatory cells and areas of fibrosis with no evidence of malignancy see in Fig. 2 and Fig. 3.

## 4 Discussion

This case posed a diagnostic challenge, as a common presentation of right lower quadrant pain led to the rare finding of both an inflammatory pseudotumor of the cecum and acute appendicitis. The clinical presentation and ultrasound findings strongly suggested acute appendicitis, supported by the patient's symptoms and elevated neutrophil count [6]. Although acute appendicitis is a common surgical emergency, this case underscores the importance of considering a broad differential diagnosis, as less common conditions can mimic its presentation [7]. The coexistence of acute appendicitis and a cecal IPT is exceptionally rare, with only a few reported cases, most of which involved the appendix rather than the cecum [[7], [8], [9]]. IPTs are benign lesions characterized by a proliferation of myofibroblastic spindle cells and inflammatory cells, and they are thought to arise from an exaggerated response to injury or inflammation [11]. The pathophysiology of IPTs is complex and not fully understood; various factors, including infectious agents, autoimmune phenomena, and genetic predisposition, have been implicated in their development [12]. The location of the IPT on the cecal wall, with a central cavity communicating with right lower quadrant fluid, is an unusual finding that suggests a localized inflammatory response.

The presence of an IPT in the vicinity of the inflamed appendix raises questions about a possible association or shared pathophysiological mechanism. It is possible that inflammation from appendicitis triggered or exacerbated an underlying IPT, or vice versa.

The patient's history of daily khat chewing is noteworthy. While khat is a stimulant with known gastrointestinal effects, its association with IPTs remains unestablished [13]. However, khat's inflammatory effects may have contributed to overall abdominal inflammation or altered the local immune response, potentially influencing the course of the IPT [12,13]. There is no evidence in literature supporting khat's role in the development or exacerbation of IPTs and further studies are needed.

This case highlights the importance of thorough surgical exploration in diagnosing rare abdominal pathologies. The intraoperative finding of the inflammatory mass prompted complete excision for histopathological examination. While IPTs are benign, they often mimic malignancy on imaging and clinical presentation, leading to diagnostic uncertainty. Differential diagnoses include malignant tumors such as sarcomas and lymphomas, adenocarcinomas, infectious masses like abscesses or granulomas (e.g., tuberculosis, fungal infections), and inflammatory conditions like Crohn's disease with mass-like inflammation. Due to the overlapping features with these conditions, IPTs are often misdiagnosed preoperatively. Surgical exploration allows for direct visualization and tissue sampling, and histopathology is essential for confirming the diagnosis of IPT, differentiating it from other potential etiologies, and guiding appropriate management. The characteristic histopathological features, including the spindle cells, inflammatory infiltrate, and absence of malignancy confirmed the diagnosis of IPT [4]. Complete surgical excision with negative margins is the preferred treatment for IPTs. Recurrence may occur if resection is incomplete [[14], [15], [16]]. While surgical resection with a negative margin is generally considered the treatment of choice, studies suggest that conservative management, including the use of corticosteroids or NSAIDs, may be effective in certain cases, particularly for IPTs in non-gastrointestinal locations. A few cases have even reported spontaneous resolution of IPTs without surgery. Given the variability in management strategies, future research is needed to establish more consistent guidelines for the treatment of cecal IPTs. While immunohistochemistry can be helpful in characterizing IPTs and differentiating them from other spindle cell lesions, it was not performed in this case. This was due to the unavailability of immunohistochemistry services in our immediate area. The nearest facility offering these services is located in Addis Ababa, the capital city Ethiopia, which is hundreds of kilometers away and would have incurred significant expenses for the patient, further limiting the affordability of this testing.

## 5 Conclusion

This case highlights a rare coexistence of acute appendicitis and an inflammatory pseudotumor (IPT) of the cecum in a young adult male. It underscores the importance of considering uncommon conditions in the differential diagnosis of acute abdominal pain and the need for thorough surgical exploration and histopathological evaluation for definitive diagnosis. The etiology of IPTs remains unclear, but this case suggests a potential link to localized inflammatory processes. Further research is essential to understand the pathophysiology of IPTs, their relationship with gastrointestinal inflammation, and optimal treatment strategies. While complete surgical excision is the standard approach, some studies explore conservative management with corticosteroids or NSAIDs, especially in non-gastrointestinal IPTs. The patient is under active follow-up with clinical evaluations and imaging to monitor for recurrence. This case emphasizes the necessity of complete excision to prevent recurrence, while also indicating that conservative management may be effective in select cases. Future research should aim to establish consistent treatment guidelines for cecal IPTs by comparing different management strategies and their long-term outcomes.

## Consent

Written informed consent was obtained from the patient for publication and any accompanying images. A copy of the written consent is available for review by the Editor-in-Chief of this journal on request. This consent was obtained after a detailed explanation of the procedure, expected outcomes, potential challenges, and possible complications was provided to the patient and his family.

## Ethical approval

The study protocol, case investigation, and consent form were thoroughly examined by the institutional review board of the College of Health Sciences at Amoud University. They granted approval for the study, along with the Ministry of Health and Borama Hospital in Awdal Region, Somaliland (BRH-201/2024). Prior to participation, written informed consent was obtained from every individual involved.

## Sources of funding

Not applicable.

## Funding

None.

## Author contribution

Dr. Abdinasir Mohamed, Dr. Ahmed Suleiman, Dr. Abdirahman Omer Ali, Dr. Hassan Elmi Moumin and Roukia Mahamad Nour individuals contributed to taking history and providing care to the patient throughout his hospital stay. Additionally, Dr. Abdinasir Mohamed, Dr. Abdirahman Omer Ali and Dr. Hassan Elmi Moumin contributed to the development of the manuscript. Dr. Isaq Omer is the pathologist.

## Guarantor

Abdirahman Omer Ali, on behalf of all authors, accept full responsibility for the work.

## Research registration number

NA

## Conflict of interest statement

The authors affirm that there are no conflicts of interest pertaining to the publication of this article.
